# Validation of the thermal stability of EMAIS-SR autonomous packaging for preservation of porcine lungs: cooling curve in an experimental model

**DOI:** 10.1590/1516-3180.2026.3638.27022026

**Published:** 2026-09-18

**Authors:** Flavio Pola dos Reis, Pedro Henrique Andrade Guimarães, Matheus Werner Fantato, Selma Ferrer da Silva, Liliane Moreira Ruiz, Natalia Aparecida Nepomuceno, Edson Caoru Kitani, Bartira de Aguiar Roza, Paulo Manuel Pêgo Fernandes

**Affiliations:** I Attending Surgeon, Thoracic Surgery Program, Instituto do Coração, Hospital das Clínicas, Faculdade de Medicina, Universidade de São Paulo (InCor-HCFMUSP), São Paulo (SP), Brazil.; II Medical Student. Faculdade de Medicina, Universidade de São Paulo (FMUSP), São Paulo (SP), Brazil.; III Medical Student. Faculdade de Medicina, Universidade de São Paulo (FMUSP), São Paulo (SP), Brazil.; IV Development Engineer, São Rafel Câmaras Frigoríficas, Arujá (SP), Brazil.; V Researcher, Laboratório de Pesquisa em Cirurgia Torácica, Departamento de Cardiopneumologia, Instituto do Coração, Hospital das Clínicas, Faculdade de Medicina, Universidade de São Paulo (InCor-HC-FMUSP), São Paulo (SP), Brazil.; VI Researcher, Laboratório de Pesquisa em Cirurgia Torácica, Departamento de Cardiopneumologia, Instituto do Coração, Hospital das Clínicas, Faculdade de Medicina, Universidade de São Paulo (InCor-HC-FMUSP), São Paulo (SP), Brazil.; VII Professor, Departamento de Eletrônica Automotiva, Faculdade de Tecnologia de Santo André (FATEC), Santo André (SP), Brazil.; VIII Associate Professor, Departamento de Enfermagem Clínica, Escola Paulista de Enfermagem, Universidade Federal de São Paulo (EPE-Unifesp), São Paulo (SP), Brazil; WHO Expert Advisory Panel, Committee on Donation and Transplantation (ECDT). Collaborating Professor PAHO-WHO, Geneva, Switzerland.; IX Full Professor, Programa de Pós-Graduação em Cirurgia Torácica e Cardiovascular, Instituto do Coração, Hospital das Clínicas, Faculdade de Medicina, Universidade de São Paulo (InCor-HC-FMUSP), São Paulo (SP), Brazil; Faculdade de Medicina, Universidade de São Paulo (HC-FMUSP), São Paulo (SP), Brazil.

**Keywords:** Models, Animals., Monitoring, Physiologic., Cold Ischemia., Organ Preservation., Experimental Study., Lung Transplantation., Thermal Stability., Controlled Hypothermic Storage., EMAIS-SR.

## Abstract

**BACKGROUND:**

Intermediate temperatures (10^°^C) better preserve mitochondrial function and extend ischemic time, which are fundamental for lung preservation during transplantation. The EMAIS-SR device was developed as a reusable, cost-effective alternative to maintain the 10^°^C set-point, thereby overcoming logistical and economic barriers in organ transport.

**OBJECTIVE:**

This study aimed to validate the thermal stability of the EMAIS-SR system in a swine model by evaluating the organ-cooling curve and the time spent within the target temperature range.

**DESIGN AND SETTING:**

Validation study using experimental surgery on porcine organs.

**METHODS:**

Lung blocks were harvested from two Landrace swine in independent experiments (P1, P2). The organs were placed in triple sterile packaging. Thermal stability was monitored for 48 h by using four negative temperature coefficient (NTC) thermistors (three intrapulmonary and one extrapulmonary) with data acquisition at a 0.1-s interval. Another datalogger (Hantek ^®^ HTM208B) was used for control and validation. Thermal distribution was further characterized by infrared thermography (FLIR C3-X).

**RESULTS:**

In Experiment 1 (P1), intrapulmonary temperatures reached the predefined target range (≤ 12^°^C) approximately 1.4 h after lung extraction, and the mean temperature recorded in the container was 8.01 ^°^C (standard deviation, 2.88^°^C). In Experiment 2 (P2), a mean temperature reduction of 6.6^°^C was observed, bringing the intrapulmonary temperature to the target range (≤ 12^°^C) within approximately 2 h post-extraction.

**CONCLUSION:**

The EMAIS-SR device effectively maintained lung-preservation temperatures within the target 8^°^C–12^°^C range over 48 h. These results demonstrate the system’s technical feasibility and its capacity to provide consistent thermal stability across intrapulmonary compartments.

## INTRODUCTION

 In the early twentieth century, Alexis Carrel demonstrated that reducing temperature to approximately 2^°^C, with the aim of reducing cellular metabolism and delaying ischemic injury, was a central strategy for preserving solid organs.^1^ Pulmonary preservation historically evolved toward static cold storage at approximately 4 ^°^C (ice).^2-3^ However, experimental studies have suggested that intermediate temperatures, particularly around 10^°^C, could offer biological advantages mainly related to mitochondrial function, although technical limitations have maintained the clinical use of the 4^°^C standard for decades.^4^

 The Toronto Lung Transplant Group revisited the investigation of lung preservation at 10^°^C, demonstrating that this temperature is optimal for preservation since it allows extension of preservation time, experimental viability, and, more recently, clinical application in human lung transplantation, with the potential to further expand preservation time.^5^ Despite these advances, the existing platforms for controlled preservation at intermediate temperatures, such as the LUNGguard^®^, are expensive and single-use, limiting their applicability in low- and middle-income countries. 

 In this context, the EMAIS-SR system has emerged as a national, reusable alternative with lower operational complexity. Originally developed for operation at 4^°^C, the device was modified for operation at 10^°^C, necessitating preclinical validation before its application in human studies. 

## OBJECTIVE

 This study aimed to validate the thermal stability of the EMAIS-SR system at 10 ^°^C in a swine model by evaluating the organ-cooling curve and the time spent within the target temperature range. 

## METHODS

 The study attempted to validate the thermal performance of the EMAIS-SR device in a swine model on the basis of experimental strategies previously adopted in animal models for organ preservation.^6^ Two independent experiments (P1 and P2) were performed with assessment of pulmonary thermal stability. All national regulations for the use of animals in research were followed, and the study was approved by the Institutional Animal Care and Use Committee (CEUA; protocol no. 2399/2026) of the Faculdade de Medicina of the Universidade de São Paulo (FMUSP). 

 Two female Landrace pigs, weighing approximately 30 kg, were used. The animals were obtained from a production unit affiliated with the Universidade de São Paulo, and all procedures were performed under veterinary supervision. The animals received premedication with intramuscular ketamine (15 mg/kg) and midazolam (0.2 mg/kg), which was followed by intravenous induction of anesthesia with propofol (7 mg/kg) and maintenance with isoflurane (1.5%–2%). After surgical access, the lung block was procured, and cold ischemia was initiated. 

 After procurement, the lungs were perfused with 3 L of isotonic saline solution (0.9% NaCl) through the pulmonary artery under continuous flow at 4^°^C. The double lung block was removed with the lung inflated to 30 cmH2O, and stapling was performed with the Panther Linear Cutter Stapler^®^. 

 The organ was placed in triple sterile packaging in accordance with Brazilian regulations: the first bag contained the lung and cold saline solution; the second bag contained saline solution; and the third outer bag was maintained dry.^7^ The packaged organ was subsequently placed in the EMAIS-SR device, which was adjusted for operation at 10^°^C.^8^

 Immediately after perfusion and at the end of the preservation period, thermal images of the lung surface were obtained using a handheld infrared camera FLIR C3-X. Thermal imaging was used as a complementary qualitative method to document the distribution of the surface temperature.^9^

 Temperatures of the lung and the preservation environment were monitored using four negative temperature coefficient (NTC) sensors, of which three were positioned within the organ (right pulmonary artery, inferior vein of the mediastinal lobe, and parenchyma of the left upper lobe) and one was placed in the container. Measurements were acquired at 0.1-s intervals with a 10-point moving average and recorded using a dedicated datalogger. In parallel, an independent datalogger (Hantek^®^ HTM208B) was placed inside the preservation container to provide external temperature monitoring for comparison and validation. Data were analyzed over a 48-h period, focusing on thermal stability and the time spent within the target range of 8^°^C–12^°^C.^10,11^

## RESULTS

 Thermal data analysis of the packaging temperature and the temperatures of the intrapulmonary compartments over 48 h of preservation was performed separately for the two independent experiments (P1 and P2). 

**Figure 1:**
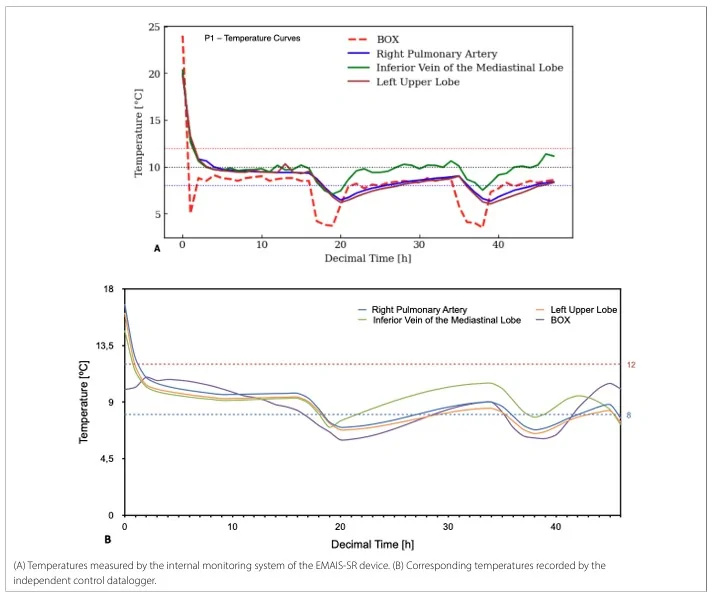
Temperature curves for the preservation container (BOX) and the intrapulmonary compartments during Experiment 1 (P1).

 In Experiment 1 (P1), intrapulmonary temperatures reached the predefined target range (≤ 12^°^C) approximately 1.4 h after lung extraction. The mean temperature reduction required to achieve this range was 5.2^°^C, which was calculated across vascular and parenchymal compartments. Initial temperatures immediately after perfusion ranged from 14.7^°^C to 17.6^°^C, and they decreased to values between 10.7^°^C and 11.6^°^C at the time of target range entry (**Figure 1A**). 

 The mean temperature recorded in the container was 8.01^°^C (standard deviation [SD], 2.88^°^C). In the right pulmonary artery, the mean temperature was 8.77^°^C (SD, 2.06^°^C) and remained within the target 8^°^C–12^°^C range for 30 h. In the inferior vein of the mediastinal lobe, the mean temperature was 9.83^°^C (SD, 1.84^°^C), and it remained within the target range for 42 h. In the parenchyma of the left upper lobe, the mean temperature was 8.6^°^C (SD, 2.16^°^C), remaining within the target range for 26 hours (**Tables 1** **and** **2**). 

**Table 1:** Descriptive temperature statistics recorded in the preservation container and in different lung regions over 48 h of preservation during Experiment 1 (P1)

**Sensor**	**Mean (^°^C)**	**SD (^°^C)**	**Min (^°^C)**	**Max (^°^C)**
BOX	8.01	2.88	3.5	24
Right pulmonary artery	8.77	2.06	6.34	19.89
Inferior vein of the mediastinal lobe	9.83	1.84	7.09	20.32
Left upper lobe	8.6	2.16	6.04	19.75

BOX, preservation container; Max, maximum temperature; Min, minimum temperature; SD, standard deviation.

**Table 2:** Cumulative time during which the temperature remained within the 8^°^ C–12^°^C range in the preservation container and in different lung regions during Experiment 1 (P1)

**Sensor**	**Time within 8^°^C–12^°^C (h)**
BOX	33
Right pulmonary artery	30
Inferior vein of the mediastinal lobe	42
Left upper lobe	26

BOX, preservation container.

 Temperature measurements obtained using the independent control datalogger are presented in **Figure 1B**. According to this control logger, the mean internal control temperature for the container (BOX) was 8.35^°^C (SD, 1.84^°^C); the mean temperature in the right pulmonary artery was 9.17 ^°^C (SD, 1.83^°^C); and mean temperatures in the inferior vein and left upper lobe parenchyma were 9.58^°^C (SD, 1.63^°^C) and 8.89 ^°^C (SD, 1.76^°^C), respectively (**Figure 1B**). 

**Figure 2:**
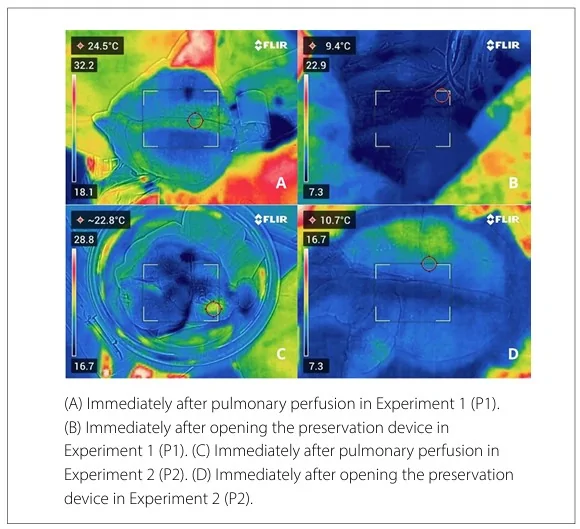
Thermal images of the lung surface.

 Qualitative thermal imaging scans obtained immediately after lung perfusion demonstrated heterogeneous surface temperature distribution, with higher temperatures (18^°^C–21^°^C) predominantly observed in the central and hilar regions (**Figure 2A**). At the end of the preservation period, thermal images acquired immediately after opening the device demonstrated a more homogeneous surface temperature distribution across the lung parenchyma, showing values consistent with the temperature range recorded by intraparenchymal sensors, within 7^°^C–12^°^C (**Figure 2B**). 

**Figure 3:**
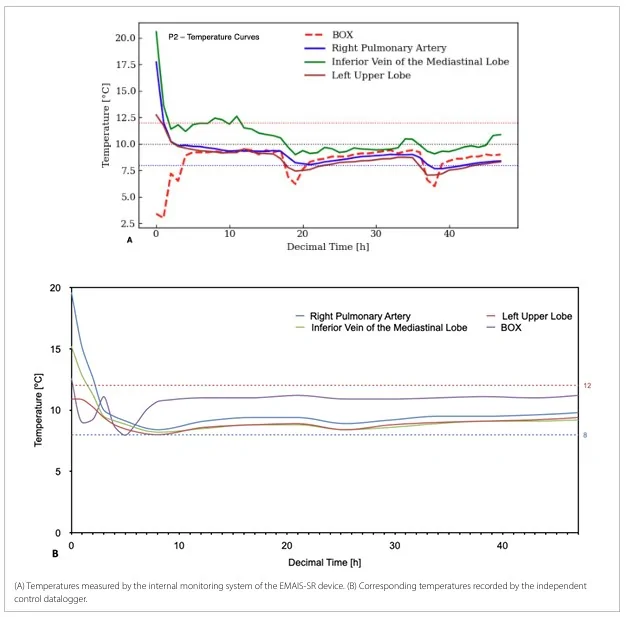
Temperature curves for the preservation container (BOX) and the intrapulmonary compartments during Experiment 2 (P2).

 In Experiment 2 (P2), intrapulmonary temperatures reached the predefined target range (≤ 12^°^C) within approximately 2 h after lung extraction. An average temperature decrease of 6.6^°^C was necessary to reach this range. Initial post-perfusion temperatures ranged from 10.9^°^C to 19.6^°^C, decreasing to values between 7.8^°^C and 10.7^°^C at the time of entry into the target range (**Figure 3A**). 

 The mean packaging temperature was 8.44^°^C (SD, 1.42^°^C) and remained within the target 8^°^C–12^°^C range for 39 h. In the right pulmonary artery, the mean temperature was 9.05^°^C (SD, 1.51^°^C) and remained within the target range for 41 h. In the inferior vein of the mediastinal lobe, the mean temperature was 10.9^°^C (SD, 2.39^°^C), and it remained within the target range for 39 h. In the left upper lobe, the mean temperature was 8.62^°^C (SD, 1.08^°^C), and it remained within the 8^°^C–12^°^C range for 33 h (**Tables 3** **and** **4**). 

**Table 3:** Descriptive temperature statistics recorded in the preservation container and in different lung regions over 48 h of preservation during Experiment 2 (P2)

**Sensor**	**Mean (^°^C)**	**SD (^°^C)**	**Min (^°^C)**	**Max (^°^C)**
BOX	8.44	1.42	3	9.5
Right pulmonary artery	9.05	1.51	7.66	17.72
Inferior vein of the mediastinal lobe	10.9	2.39	8.98	20.58
Left upper lobe	8.62	1.08	7.04	12.73

BOX, preservation container; Max, maximum temperature; Min, minimum temperature; SD, standard deviation.

**Table 4:** Cumulative time during which the temperature remained within the 8^°^C–12^°^C range in the preservation container and in different lung regions during Experiment 2 (P2)

**Sensor**	**Time within 8^°^C–12^°^C (h)**
BOX	39
Right pulmonary artery	41
Inferior vein of the mediastinal lobe	39
Left upper lobe	33

BOX, preservation container.

 Temperature measurements obtained using the independent control datalogger are registered in **Figure 3B**. According to this device, the mean internal temperature for the container (BOX) was 10.66^°^C (SD, 0.82^°^C), and the mean temperature in the right pulmonary artery was 9.77^°^C (SD, 1.68^°^C). The mean temperatures in the inferior vein of the mediastinal lobe and the left upper lobe were 9.01^°^C (SD, 1.05^°^C) and 8.84^°^C (SD, 0.61^°^C), respectively (**Figure 3B**). 

 For Experiment 2 (P2), thermal imaging performed immediately after pulmonary perfusion showed elevated surface temperatures mainly distributed over the central lung regions, with values ranging from 20^°^C to 23^°^C, while the peripheral areas exhibited lower temperatures (**Figure 2C**). After completion of the preservation period, thermal images obtained showed values predominantly within the 7^°^C–12^°^C range, in agreement with intraparenchymal temperature recordings (**Figure 2D**). 

## DISCUSSION

 This pilot study demonstrated that the EMAIS-SR device, adjusted to operate at 10^°^C, was able to maintain lung-preservation temperatures within the predefined range of 8^°^C–12^°^C during a 48 h preservation period in a swine model. Temperature measurements obtained from the preservation container and from intrapulmonary vascular and parenchymal sites showed concordant behavior over time, without sustained deviations outside the target range. These findings demonstrate the thermal stability of the EMAIS-SR system for a biological model and support its use in subsequent studies involving human lungs.^9,11,12^

 An additional relevant finding was the time required for the organ to reach the target temperature range after procurement. In the present study, the EMAIS-SR device achieved temperatures below 12^°^C within 1.7 h (range, 1.4–2 h) after lung procurement. Rapid lung cooling has not been suggested to be associated with structural or functional injury. In contrast, abrupt rewarming during transplantation can trigger pro-inflammatory cascades, metabolic dysregulation, and oxidative stress, thereby limiting the effective cold ischemia time of the lung.^13^ In this context, the EMAIS-SR system was designed to prevent unintended temperature elevations through active thermal control mechanisms. 

 Controlled hypothermic-preservation devices have been increasingly shown to outperform conventional ice-based storage in lung transplantation.^9,11,12^ Comparative studies have demonstrated that devices with active temperature control allow superior maintenance of mitochondrial integrity and cellular viability than ice-filled containers.^5,14,15^ Within this scenario, the EMAIS-SR system has emerged as a reusable alternative for lung transport. Importantly, previous studies have demonstrated effective microbiological decontamination of the device, supporting its safe reuse and addressing a key limitation of the existing disposable systems.^7^ This aspect is particularly relevant in low- and middle-income settings, where the high cost of single-use devices limits their widespread adoption and perpetuates reliance on conventional ice-based preservation. 

 The cooling dynamics of the EMAIS-SR system involve intermittent and controlled temperature drops to maintain the 10^°^C set-point. These fluctuations are visible as "valleys" in the NTC sensor graphs, demonstrating that the lung parenchyma tracks ambient thermal variations more slowly due to its mass and insulating properties. Despite these oscillations, thermal variation remained within the 8^°^C–12^°^C stability range specified in the experimental protocol. The literature still lacks standardized and universally accepted methods for real-time temperature verification.11 The difficulty in obtaining absolute measurements of parenchymal temperature is evidenced by the differences observed between the internal monitoring system (NTC) and the independent control datalogger (Hantek^®^ HTM208B). In Experiment 1 (P1), modest variations were observed, such as in the right pulmonary artery (8.77^°^C with the NTC versus 9.17^°^C with the control datalogger) and in the left upper lobe parenchyma (8.6^°^C versus 8.89^°^C). In Experiment 2 (P2), these disparities were more pronounced, with the internal system recording a container temperature of 8.44^°^C and the control datalogger recording a container temperature of 10.66^°^C, and inferior vein temperatures of 10.9^°^C versus 9.01^°^C. Anticipating this difficulty, the present study adopted multiple measurement methods (container sensors and external control), allowing for cross-validation that more closely approximates the organ’s true thermal reality. 

 Preservation at temperatures around 10^°^C, in comparison with the current gold standard of 4^°^C (ice), has been consistently supported by experimental studies. These studies have demonstrated that prolonged graft preservation at 10^°^C ensures superior oxygenation and lung compliance while reducing airway pressures and edema, which is characterized by organ weight gain during ischemia. Furthermore, microscopic analyses have shown enhanced preservation at 10^°^C, evidenced by higher accumulation of the cytoprotective metabolites itaconate, glutamine, and N-acetylglutamine, which activate the innate mitochondrial antioxidative system that is essential for maintaining organelle health.15 In addition to experimental data, clinical studies have supported the adoption of preservation at 10^°^C, demonstrating increased preservation time and a consequent extension of the cold ischemia period. Clinically, this approach indicates a reduced incidence of primary graft dysfunction (PGD), the leading cause of early mortality following lung transplantation.^5^

 Thus, the capacity of the EMAIS-SR system to maintain thermal stability within the 10^°^C range, combined with its reusability, represents a promising advancement for lung transplant logistics. This system can replace the current gold standard of ice-based transport, which has been shown to be inferior due to its inability to maintain the organ within the ideal range for viability, furthering the development of lung transplantation techniques.^16-18^

 Despite its promising results, this study had some limitations inherent to its experimental and pilot design. Preservation was performed using isotonic saline rather than a preservation solution, and the number of animals was limited for financial and ethical reasons. Furthermore, the observed differences between the measurement methods used in this study reinforce the need to develop more effective and precise thermal-monitoring tools and methods in lung preservation. Moreover, no histological or functional analyses were performed, because the primary objective of the study was to characterize the thermal behavior of the system. 

 These aspects will be addressed in subsequent studies, including investigations using human lungs declined for transplantation, wherein thermal stability can be correlated with morphological, metabolic, and functional parameters. 

## CONCLUSION

 In this preclinical swine study, the EMAIS-SR device demonstrated the capacity to maintain lung-preservation temperatures predominantly within the target range of 8^°^C–12^°^C over a 48 h period. Thermal stability was observed in the preservation container and reflected across intrapulmonary compartments on the basis of continuous temperature monitoring. These findings support the technical feasibility of the system for lung preservation at intermediate temperature and support its progression to further translational evaluation. 

## Data Availability

Data supporting the findings of this study are available upon request from the corresponding author, Flavio Pola dos Reis.
